# Consumption Patterns of Sugar-Sweetened Beverages and Association with Undernutrition among Children Aged 9–17 Years in Guangzhou, China: A Cross-Sectional Study

**DOI:** 10.3390/nu16050650

**Published:** 2024-02-26

**Authors:** Jiaying Guo, Shiyun Luo, Zheng Su, Jinhan Fu, Jie Ma, Xuexin Zhong, Chunzi Zeng, Jie Huang, Weiwei Zhang, Zhoubin Zhang, Huilian Zhu, Yan Li

**Affiliations:** 1 School of Public Health, Sun Yat-sen University, Guangzhou 510080, China; guojy78@mail2.sysu.edu.cn (J.G.); fujh6@mail2.sysu.edu.cn (J.F.); zhongxx27@mail2.sysu.edu.cn (X.Z.); zhuhl@mail.sysu.edu.cn (H.Z.); 2 Department of Foodborne Diseases and Food Safety Risk Surveillance, Guangzhou Center for Disease Control and Prevention, Guangzhou 510440, China; luoshy25@mail3.sysu.edu.cn (S.L.); gzcdc_zengcz@gz.gov.cn (C.Z.); huangjie1026@126.com (J.H.); gzcdczhangww@foxmail.com (W.Z.); gzcdczzb@gzcdc.org.cn (Z.Z.); 3 School of Public Health, Southern Medical University, Guangzhou 510515, China; sue1995@smu.edu.cn (Z.S.); jasmine225@smu.edu.cn (J.M.)

**Keywords:** sugar-sweetened beverages consumption patterns, undernutrition, children, China

## Abstract

Globally, the high consumption levels of sugar-sweetened beverages (SSBs) and their effect on health have drawn significant attention. This study aimed to identify the consumption patterns of SSBs among children in rural areas of Guangzhou, China, and explore their association with undernutrition. A total of 1864 children aged 9–17 years old were included in this study. Demographics, lifestyle behaviors, and anthropometric and dietary information were collected. Factor analysis was used to identify patterns of SSBs, while nutritional status was assessed using Body Mass Index (BMI). Latent class analysis was used to establish dietary preference models. Log-binomial regression analysis was used to analyze the association between SSBs consumption patterns and undernutrition. The undernutrition prevalence in children was 14.54–19.94% in boys and 9.07% in girls. Three SSB consumption patterns were identified, including the plant protein pattern, dairy-containing pattern, and coffee pattern. Both medium-high (Q3) and the highest (Q4) scores in the dairy-containing pattern were positively associated with the risk of undernutrition, especially in boys. Furthermore, the highest scores in the plant protein pattern and coffee pattern were positively associated with the risk of undernutrition in children aged 9–10 years old. The dairy-containing pattern was a risk factor for undernutrition in children, especially for boys; the plant protein patterns and coffee patterns were risk factors for undernutrition in children aged 9–10 years old. The findings of the study can provide scientific evidence and policy recommendations for improving children’s health conditions.

## 1. Introduction

Undernutrition includes wasting and stunting [1] and is one of the important public health issues affecting global children’s health. Childhood undernutrition not only impacts the skeletal and brain development of children but also increases the risk of developing various diseases [2,3]. In 2022, 22.3% (148 million) of children under the age of five worldwide were stunted, and 6.8% (45 million) were wasted [4]. In China, the prevalence of undernutrition among Han ethnic students aged 7–18 was found to be 8.64% [5]. A recent study of the “Rural Compulsory Education Student Nutrition Improvement Plan” in 2021 revealed that the detection rate of undernutrition among students aged 6–15 years old was 12.1% [6]. These findings indicate that undernutrition in children still needs attention in China, particularly in rural areas.

There are many factors influencing undernutrition, such as the economic status and sanitation conditions of the residential area, dietary characteristics determined by the geographic location, and so on [1]. As the consumption of SSBs remains high globally, numerous studies have reported that the excessive intake of SSBs significantly impacts the nutritional status of children, such as overweight, obesity, hypertension [7], and cardiometabolic disease [8]. However, the studies on the link between SSBs and undernutrition were limited [9]. Recent studies have suggested that the consumption of SSBs may be associated with the inadequate intake of micronutrients and poor-quality diets, which are all potential risk factors for undernutrition in children [10,11]. Substituting a regular dietary intake with sugary liquids has been reported to lead to significant and sustained weight loss [12]. The beverage type was found to be a determining factor in short-term food intake suppression in boys [13]. Caffeine intake and a high consumption of SSBs have been linked to an increased risk of childhood osteoporosis and fractures, which are markers of undernutrition in bone development [14,15]. Notably, the behaviors of SSBs consumption are correlated with children’s undernutrition. However, no study has explored this issue from the perspective of SSBs consumption patterns, which involve multiple dimensions, including the type of beverage, the composition of nutrients, and the amount consumed.

Guangzhou is located in the southern region of China with a hot climate, and children have a greater demand for beverages, water, etc. Therefore, this study aims to explore the association between SSBs consumption patterns and undernutrition among children in rural areas of Guangzhou, China. We aim to fill the existing research gaps, provide new insights into understanding the issue of childhood undernutrition, and thereby offer scientific evidence and policy recommendations for improving children’s health conditions.

## 2. Materials and Methods

### 2.1. Participants

The cross-sectional study was conducted from April 2021 to June 2023. Participants were recruited using a multistage stratified cluster random sampling method: (1) Random selection of four primary schools, five middle schools, and two high schools from rural areas in Guangzhou. (2) Employing a stratified sampling approach involving the random selection of two to three grades from each primary school, two grades from each junior high school, and one grade from each senior high school. (3) Random selection of two to three classes of students from each grade.

**Figure 1:**
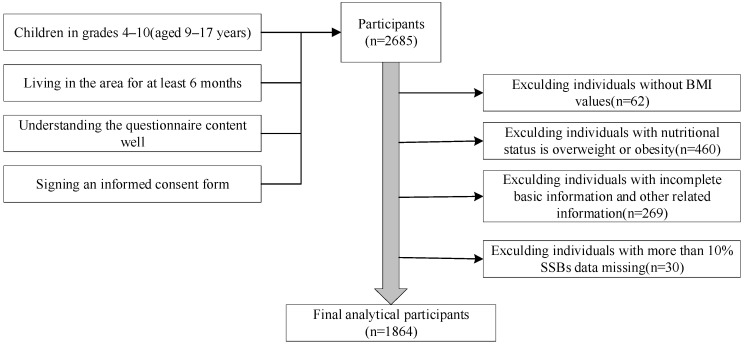
Selection process flowchart for research participants.

The sample size calculation was based on the following formula: N = deff$\frac{u_{\frac{\alpha }{2}}^{2}P(1-P)}{\delta^{2}}\;$ [16]. The parameters were as follows: 95% confidence level and $u_{\alpha /2}$ = 1.96. Based on the 2019 detection rate of undernutrition among compulsory education children in Guangzhou [17], the probability P was 8.70%. The design effect (deff) was set at 1.5; the relative error (*r*) was 20%; and δ was calculated as 20% × 8.7%. This computation resulted in a sample size of 1512 students. The actual sample size was expanded by 10% due to the invalid questionnaires and refusal rates taken into account. A minimum of 1663 students were required for the survey. The process for selecting survey respondents is detailed in Figure 1.

A total of 2685 students were investigated in this study. According to the research objectives, only normal and undernutrition populations were included for analysis, resulting in a sample size of 2225 students. Cases with missing body measurement data and other related information were excluded, leaving 1864 students for the final analysis.

### 2.2. Survey Content

In this study, face-to-face interviews were conducted with children by uniformly trained research assistants. The survey comprised two main components: a questionnaire and a physical examination.

(1) Questionnaire survey: (1) Information on demographics covered age, gender, region, place of residence, and parent’s education level, among others. (2) Lifestyle variables included moderate-to-high physical activity, screen time, dietary preferences, nutrition knowledge level, etc. (3) The frequency and intake of SSBs among students in the past month were assessed using a semi-quantitative food frequency questionnaire (FFQ). Visual aids such as food models and images were employed to assist participants in evaluating their SSB intake. The questionnaire was developed based on the survey conducted by the China National Center for Chronic Noncommunicable Disease and Nutrition Surveillance [18] and further adjusted through group discussions considering the characteristics of Guangzhou children. SSBs were classified into nine categories according to the Chinese General Principles for Beverages (GB/T 10789-2015) [19]: carbonated beverages, fruit and vegetable juices and their beverages, plant protein beverages, dairy-containing beverages, tea (and its types) beverages, coffee (and its types) beverages, plant-based beverages, milk tea beverages, and sports beverages.
(2) Physical examination: Physical assessments such as height and weight were performed meticulously [20] using a mechanical height meter and an electronic scale, accurate to 0.1 cm and 0.1 kg, respectively. Body mass index (BMI) was computed as the ratio of weight in kilograms to the square of height in meters (BMI = weight (kg)/height (m^2^)).

### 2.3. The Criteria for Diagnosing Undernutrition

The nutritional status was assessed using the “Screening Standards for Undernutrition in School-Aged Children and Adolescents” (WS/T456-2014), which is a national health industry standard promulgated in 2014 [21]. According to this standard, individuals with a height less than or equal to the gender-age group’s “stunting” threshold were classified as stunting. After excluding those with stunting, individuals with a BMI less than or equal to the gender-age group’s “moderate to severe wasting” threshold were classified as having moderate to severe wasting, while those with a BMI within the gender-age group’s “mild wasting” threshold range were classified as having mild wasting.

### 2.4. The Establishment of Sugar-Sweetened Beverages Consumption Patterns

The establishment of SSBs patterns was conducted using exploratory factor analysis (EFA). The details of this analysis have been described more comprehensively previously [22]. In this study, a KMO test with a result greater than 0.78 and a Bartlett’s sphericity test with a significant *p*-value (*p* < 0.001) were applied. Components of SSBs patterns were determined by retaining factors with absolute factor loadings greater than 0.5 [23].

### 2.5. The Establishment of Dietary Preferences Models

The establishment of a dietary preferences model was achieved through latent class analysis (LCA). The principle of latent class analysis is based on parameter estimation using the response patterns of individuals on observed indicators, which are represented by different joint probabilities. Two important parameters in this analysis are the latent class probability (which explains the proportion of individuals in each category) and the conditional probability (referring to the probability that an individual within a latent class responds positively to the observed indicator) [24].

In this study, behaviors related to the frequency of consuming desserts, fried foods, snacks, western fast food, fresh fruits, vegetables, dairy products, and breakfast were examined. Individuals who consumed these foods less than 3 times per week were considered infrequent consumers and assigned a value of 0, while those who consumed them 3 or more times per week were considered frequent consumers and assigned a value of 1.

The LCA model with one category was initially fitted, and then the number of categories was gradually increased. The Expectation-Maximization (EM) algorithm based on maximum likelihood estimation was used for parameter estimation and the calculation of model fit evaluation metrics in models with different numbers of categories [25]. The Aikake information criterion (AIC), Bayesian information criterion (BIC), adjusted Bayesian information criterion (aBIC), lo-Mendel-Rubin adjusted likelihood (LMR), likelihood ratio test based on Bootstrap (BLRT), and Entropy values were comprehensively considered.

### 2.6. Statistical Analysis

The questionnaire was sorted and coded uniformly. Double entry of the data was carried out using EpiData version 3.1 and created the initial database. The integrity and logical consistency of the data were verified using Microsoft Excel 2019. Categorical variables were represented as *n* (%) in the data description. Factor analysis was used to construct SSBs consumption patterns, and the dietary preferences model was established using latent class analysis. Categorical variables were compared using chi-square tests and the chi-square trend test.

A cross-sectional design was used in this study, and the prevalence of undernutrition exceeded 10%. To avoid the potential overestimation of the relationship between undernutrition and independent variables that could occur with odds ratio reporting, prevalence ratios were deemed the most appropriate measure of association. Hence, log-binomial regression analysis was conducted to identify predictive factors for undernutrition. Prevalence ratios (PR) and 95% confidence intervals (CI) were calculated, along with the estimation of linear trends in PR. Two models were fitted for each SSBs consumption pattern: one unadjusted and one adjusted for covariates. Model 1 represents the unadjusted analysis. Model 2 incorporated additional factors based on the survey and literature data, including gender, age, mother education level, boarding status, dietary preferences [25], and nutritional knowledge level [26].

The screen time was divided into two categories according to the requirements of the “Physical Activity Guidelines for Chinese Population (2021)” [27]: less than or equal to 2 h per day and more than 2 h per day. The level of nutritional knowledge was assessed using a scoring system; the median score was used as the threshold value [26] for screening qualified and unqualified individuals.

Gender and age were selected as stratification variables. The age stratification was based on “Chinese Residents’ Dietary Guidelines (2022)” for school-aged children [28]. The statistical description and inference were conducted using SPSS 26.0 and Mplus 8.3, with a significance level set at *p* < 0.05 (two-tailed). Data visualization was performed using Graph Pad Prism 8 and Microsoft Excel 2019.

## 3. Results

### 3.1. Participant Characteristics

This study encompassed 1864 children with complete data (50.32% male, comprising 938 individuals; 49.68% female, comprising 926 individuals) (Table 1), aged between 9 and 17 years, with a mean age of 13.18 years. The rate of undernutrition was 14.54–19.94% in boys and 9.07% in girls. Compared to the normal group, children with undernutrition were more likely to be 9–10 years old, be male, have a mother with lower levels of education, be not boarding, and have inadequate knowledge of nutrition (*p* < 0.05).

### 3.2. Consumption Patterns of Sugar-Sweetened Beverages

**Figure 2:**
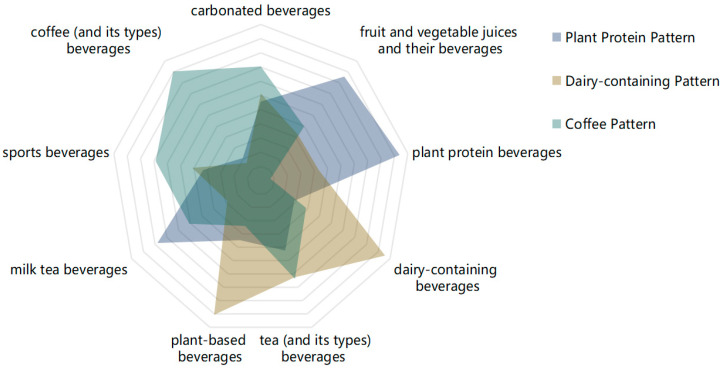
Radar chart depicting the various beverages patterns derived from factor analysis.

The presentation of SSBs patterns determined through principal component analysis is shown (Figure 2, Table 2). Factor analysis identified three main SSBs consumption patterns from the nine SSBs, explaining 16.43% (plant protein pattern), 16.11% (dairy-containing pattern), and 15.27% (coffee pattern) of the variance, collectively explaining 47.81% of the total variance. The plant protein pattern mainly includes plant protein beverages, fruit and vegetable juices and their beverages, and milk tea beverages. The dairy-containing pattern mainly includes dairy-containing beverages and plant-based beverages. The coffee pattern mainly includes coffee (and its types) beverages and carbonated beverages.

### 3.3. Latent Class Model of Other Dietary Preferences

Model fitting was performed using eight variables, such as the frequency of consumption of desserts, fried foods, snacks, fast food, fresh fruits, vegetables, dairy products, and breakfast. Five latent class models were evaluated. The criteria for assessing the fit of these models included: (1) lower AIC, BIC, and aBIC values indicating a better model; (2) entropy values closer to one, denoting a more accurate classification [29]; (3) significance in the BLRT and the LMR tests (*p* < 0.05), suggesting the model with “n” categories is superior to the model with “*n* − 1” categories [24]. A comparison of the five models revealed that although the four-category model had the smallest AIC, BIC, and aBIC, its LMR was not significant (Table 3). The two-category model showed higher entropy, and both the BLRT and LMR were significant, indicating its superiority over the three-category model. After comprehensive consideration, the two-category model was chosen as optimal.

**Figure 3:**
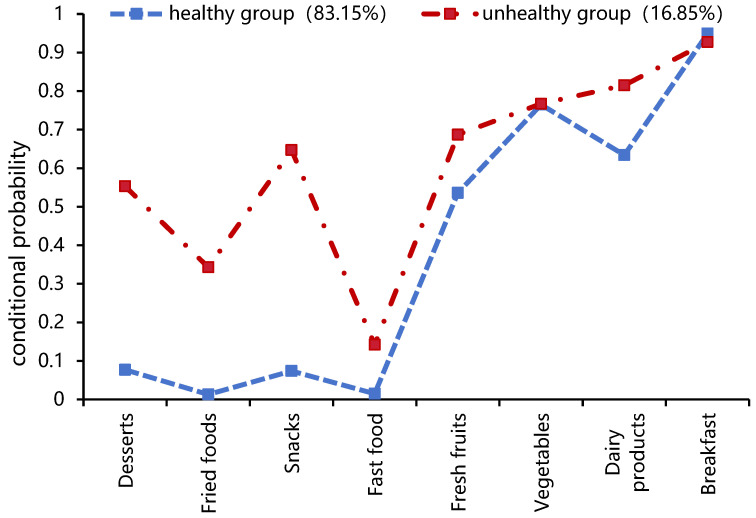
Conditional probability distribution of two potential categories.

Based on the conditional probability distribution of the eight dietary behaviors within the two identified profiles, the categories were named (Figure 3). In Category 1, individuals with high probabilities of frequently consuming vegetables and fruits and low probabilities of consuming sweets, fried foods, snacks, and fast food were defined as the “Healthy Group”. Category 2, characterized by high probabilities of dessert, fried food, snack, and fast food intake, was labeled the “Unhealthy Group”. The estimated probabilities for each of the two groups are 83.15% (Healthy Group) and 16.85% (Unhealthy Group), respectively.

### 3.4. Characteristics of Quartiles (Q) of SSBs Consumption Patterns in Study Participants

The characteristics of the Q1 (lowest) and Q4 (highest) quartiles for the three SSBs patterns are shown (Table 4). The analysis revealed that children who had dietary preferences in the unhealthy group were associated with all three SSBs patterns. Children aged 14–17 years who live in a boarding school were more likely to align with the plant protein pattern. Boys who engage in high-intensity physical activity (equal to or more than three times per week) were more likely to fit into the dairy-containing pattern. Children in the highest quartile of the coffee pattern were more likely to be boys, and those aged 14–17 years engage in high-intensity physical activity (equal to or more than three times per week) and spend a longer time on screen time per day (more than 2 h per day).

### 3.5. Association Analysis between SSBs Consumption Patterns and Undernutrition

#### 3.5.1. Analysis of SSBs Consumption Patterns and Undernutrition

The level of preference for the dairy-containing pattern was found to have a linear relationship with the risk of undernutrition (Table 5, *p* < 0.001 R = 0.086, *p* < 0.001). It suggested that individuals with a higher preference for the dairy-containing pattern have a higher risk of undernutrition.

After adjusting for gender, age, mother’s education level, boarding, dietary preferences, and nutritional knowledge level, the medium-high-tendency (Q3) and highest-tendency groups (Q4) of the dairy-containing pattern were observed to have a significantly higher risk of undernutrition in comparison to the lowest-tendency group (Q1) (PR = 1.410, 95% CI: 1.010~1.968, *p* = 0.043) (PR = 1.506, 95% CI: 1.088~2.083, *p* = 0.014).

#### 3.5.2. Log-Binomial Regression Analysis on Undernutrition in Children of Different Genders and Ages Based on SSBs Patterns

**Figure 4:**
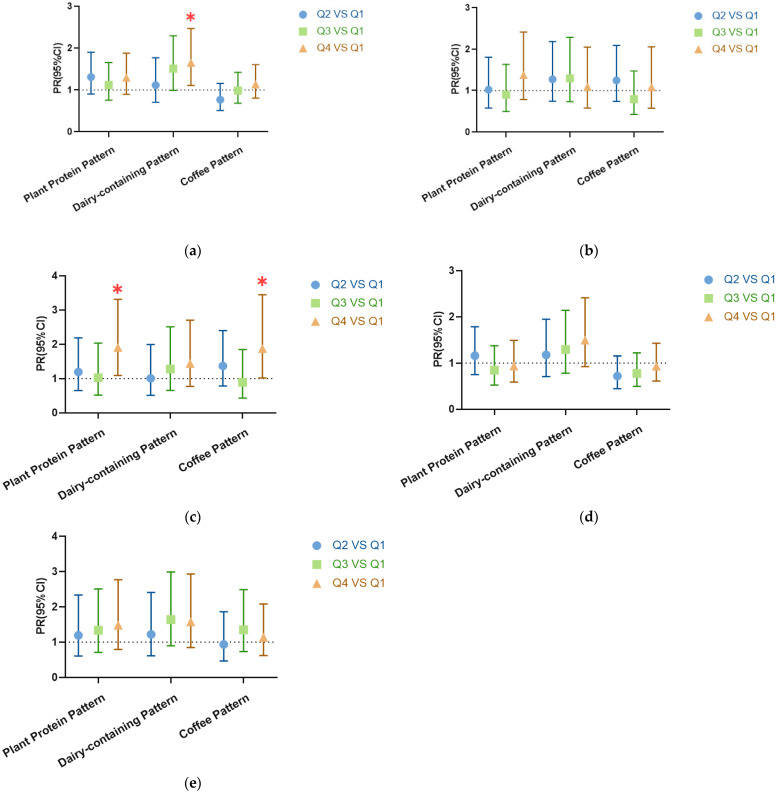
Log-binomial regression analysis was used for the analysis. Prevalence ratio and 95% CI in the second (Q2), third (Q3), and highest (Q4) groups of the three sugar-sweetened beverages consumption patterns compared to the lowest (Q1). (**a**) Males, *n* = 938; (**b**) females, *n* = 926; (**c**) children aged 9–10, *n* = 335; (**d**) children aged 11–13, *n* = 909; (**e**) children aged 14–17, *n* = 620. All models were adjusted for gender, age, mother’s education level, boarding status, dietary preferences, and nutritional knowledge level. “∗” mean *p*-value < 0.05. A detailed table is in the Supplementary Materials.

The results of the stratified analysis are presented (Figure 4). After stratifying by gender, boys in the dairy-containing pattern with the highest quartile (Q4) were more likely to experience undernutrition compared to those in the lowest quartile (Q1) (PR = 1.654, 95% CI: 1.108~2.469, *p* = 0.014) (Figure 4a).

Upon further stratification by age, children aged 9–10 years old in the plant protein pattern and coffee pattern with the highest quartile (Q4) were more likely to experience undernutrition compared to those in the lowest quartile (Q1) (PR = 1.901, 95% CI: 1.091~3.313, *p* = 0.023) (PR = 1.873, 95% CI: 1.018~3.446, *p* = 0.044) (Figure 4c).

## 4. Discussion

Dietary habits and diet-related risk factors are regarded as significant contributors to global mortality and morbidity [30]. In 2016, approximately 10% of the global disease burden was linked to diets low in nutritional value [31]. Among the various dietary risk factors, SSBs have received particular attention. SSBs are characterized as energy-dense and nutrient-poor, containing various forms of added sugars [32]. Data from the National Health and Nutrition Examination Survey (NHANES) and research indicate that SSBs are primarily consumed by adolescents across all age groups [33,34]. The current situation in China has a similar trend; the proportion of children aged 6–17 years who frequently (more than five times per week) consume SSBs stands at 18.9% nationwide, with 21.2% in urban areas and 16.7% in rural areas [35]. The intake of SSBs can affect the consumption volumes of food groups such as fruits, vegetables, and dairy and then impact the intake of various nutrients and produce adverse effects. A high tendency to consume SSBs is associated with an increased risk of multiple nutrition-related diseases, including a low bone mineral density [36], hypertension [7], trace mineral deficiency, and cardiometabolic diseases [8], thus contributing to the global disease burden [37].

In China, school-aged children face the double burden of undernutrition and overnutrition [38]. Our previous research [17] showed that in 2019, the overall detection rate of undernutrition among primary and secondary school students in Guangzhou was 8.7%. In the present study, the undernutrition prevalence in children in rural areas of Guangzhou was 14.54%. Among the many types of undernutrition, most children exhibit short-term undernutrition, which is due to an inappropriate dietary intake [39]. Undernutrition is a serious threat to the health of children. Many factors lead to undernutrition in children; in view of the high consumption of SSBs being among them, we focused on exploring the impact of SSBs on undernutrition.

In this cross-sectional study, three SSB consumption patterns were identified, including the plant protein pattern, dairy-containing pattern, and coffee pattern. The three SSBs consumption patterns identified in this study reflect the beverage preferences among school-aged children in rural areas of Guangzhou. For instance, the dairy-containing beverage pattern encompasses beverages that are often commonly misconstrued as healthy choices. These results can also provide a reference for subsequent dietary guidance. Moreover, compared to a single SSBs intake, SSBs consumption patterns can more comprehensively reflect children’s overall beverages intake [40], while compared to the total number of SSBs, the consumption patterns can more accurately reflect the distinct characteristics and varying effects on undernutrition of different SSBs.

In this study, after adjusting for confounding factors, the tendency level of the dairy-containing pattern was positively associated with the risk of undernutrition. The most prominent feature of the dairy-containing pattern was a high intake of dairy-containing beverages and plant-based beverages. Dairy-containing beverages refer to protein drinks processed or fermented using milk and/or dairy products, with the addition or exclusion of other food ingredients and/or food additives [41]. Due to its sweet and sour taste, dairy-containing beverages are popular among children [42]. Prior research has identified that dairy-containing beverages can reduce the intake of core foods such as fruit and vegetables, affecting the intake of micronutrients and leading to potential deficiencies in a variety of micronutrients in children’s diets [11]. Meanwhile, the whey protein derived from dairy products has a stronger inhibitory effect on food intake. It could curb short-term eating, increase fullness, and activate satiety-signaling mechanisms [43]. Protein-induced appetite suppression may be attributed to bioactive peptides and amino acids formed during digestion. Whey protein and casein could also enhance the release of satiety hormones like cholecystokinin [43]. Similarly, plant-based beverages generally contain some medicine and food homologous to traditional Chinese medicine [44], such as chrysanthemum, which some parents think has certain health functions. However, some plant-based beverages contain certain amounts of cellulose, which might cause gastrointestinal discomfort and affect the digestion and absorption of nutrients.

The gender-stratified analysis revealed that boys with a high tendency level of the dairy-containing pattern had a high risk of undernutrition, potentially due to differing nutritional sensitivities between genders [45]. Existing studies have shown that beverage type is a determinant factor in short-term food intake suppression in boys [13]. The survey showed that dairy-containing beverages have lower added sugar (7.9 g/100 mL) and higher protein (≥1.0 g/100 g) than other types of SSBs [23]. It has been demonstrated that glucose can inhibit food intake in boys aged 9–14 years [46], and a whey protein derived from dairy products has a stronger inhibitory effect on the food intake of healthy-weight boys compared to glucose [13,47]. During late adolescence, boys undergo significant growth, requiring increased energy intake. Even after adjusting for body fat, lean body mass, height, and weight status, boys still consume more energy than girls [48]. One study [17] indicated a greater undernutrition risk in boys than in girls. The appetite suppression from dairy-containing beverages may lead to an insufficient energy intake and a higher undernutrition risk among boys.

After stratifying for age, the tendency level of the plant protein pattern and coffee pattern was positively associated with the risk of undernutrition in children 9–10 years old. This result is consistent with the study by Dong Yanhui, which showed that the age bracket of 9–11 years is particularly susceptible to undernutrition [49]. There may be two possible reasons for this phenomenon. First, the plant-protein pattern and coffee pattern could be linked to hormonal changes in children aged 9–10 years. A study involving children aged 6–9 years found a negative correlation between SSBs and dehydroepiandrosterone sulfate (DHEAS) after adjusting for gender, age, and body fat percentage [50]. DHEAS serves as a precursor to androgens, such as testosterone and dihydrotestosterone, which contribute to the clinical signs of adrenarche. Adrenarche potentially boosts the adrenal cortex function, facilitating both height and weight gain [51]. Second, the plant-protein pattern and coffee pattern might reduce the appetite of children aged 9–10 years. After consuming SSBs like milk tea and carbonated beverages, the sugar in the beverages can increase blood sugar levels and decrease the ghrelin response [52]; then, the demand for food will be reduced [53]. Research has shown that young children have stronger and more precise responses to hunger and satiety signals to regulate energy intake compared to older children [54], and this compensation accuracy seems to decline with age [55].

The characteristic of the plant-protein pattern was a high intake of plant-protein beverages, fruit and vegetable juices and their beverages, and milk tea beverages, all of them containing a lot of added sugars. Consuming a large amount of added sugar can modify the dopaminergic reward system in the central nervous system, resulting in pleasure responses [56,57]. This can potentially lead to a consistent increase in SSBs consumption and a preference for sweet tastes [58]. Habituation to intense sweetness could diminish the taste appeal and attractiveness of naturally sweet, healthy foods such as apples or carrots [59]. This could potentially disrupt the oral microbiota during childhood and cause bacterial disturbance and dental problems [11], resulting in a poor-quality diet.

The coffee pattern includes carbonated beverages and coffee (and its types) beverages, both of which contain caffeine. In accordance with the recommendations from the American Academy of Child and Adolescent Psychiatry, children under 12 years old are advised to avoid any caffeine consumption [60]. The brains of children are not fully developed, and the intake of stimulants may lead to some long-term effects. One important cause of undernutrition caused by caffeine may be its impact on sleep. The nighttime secretion of the growth hormone relies on sleep [61]. Studies have found that among lean children aged 10–11, prolonged sleep is beneficial for height growth and weight gain [62]. Conversely, shortened or poor-quality sleep not only affects children’s growth and development but also correlates significantly with an increased intake of sugar and SSBs [63,64]. This correlation establishes a vicious cycle. The other important cause of undernutrition caused by caffeine may be its impact on metabolism. Caffeine stimulates the central nervous system [65] and increases metabolism in the human body. Children tend to be more sensitive to caffeine [66], and long-term intake can lead to an increased body expenditure and negative energy balance, which may develop into undernutrition. In addition, carbonated beverages like colas contain phosphoric acid, and the excessive ingestion of external phosphates is considered to disrupt vitamin D and calcium metabolism [67], resulting in a negative impact on children’s bone density and harm to children’s height growth [14,15].

This study also found that children with unhealthy dietary preferences characterized by the high-frequency consumption of sweets and fried foods had a high propensity for all SSBs consumption patterns. This is consistent with research by Lu Shuang et al. [25]. This could be due to the high intake of SSBs increasing the demand for sweetness, which in turn leads to the consumption of higher-calorie foods. In real life, the combination of SSBs with other high-calorie foods is more appealing to children than pairing SSBs with healthy foods. Notably, boarding school children aged 14–17 years were more inclined toward the plant protein pattern, potentially due to the social effects of milk tea beverages. Older boarding students spend more time at school and with peers, and milk tea beverages have become a “social currency” among young people that aligns with trends and peer approval [68].

This study has three main limitations. First, this is a cross-sectional study, so causality between SSBs consumption and undernutrition cannot be established; the possibility of reverse causation exists, and further study is needed. Second, the study utilized questionnaires to collect data on lifestyle behaviors and beverages intake; recall bias and social desirability bias cannot be avoided. Third, the participants of this study were children from rural areas of Guangzhou; hence, the results need to be extrapolated with caution due to potential population differences.

## 5. Conclusions

In conclusion, the association between sugar-sweetened beverages and undernutrition remains an issue of concern. This study identified three SSBs consumption patterns, including the plant protein pattern, dairy-containing pattern, and coffee pattern. The study indicated that the association between different SSBs consumption patterns and undernutrition varies across genders and age groups in adolescents. The dairy-containing pattern was a risk factor for undernutrition in children, especially for boys; the plant protein pattern and coffee pattern were risk factors for undernutrition in children aged 9–10 years old. The findings of the study can provide ideas for nutritional intervention and improvement in adolescents.

## Figures and Tables

**Table 1:** Demographic and lifestyle characteristics of the study participants.

Variable	Total	Nutrition Status		*p*
		Normal	Undernutrition	
Age, *n* (%)				
9–10	335	268 (80.00)	67 (20.00)	**0.007**
11–13	909	785 (86.36)	124 (13.64)	
14–17	620	540 (87.10)	80 (12.90)	
Sex, *n* (%)				
Male	938	751 (80.06)	187 (19.94)	**<0.001**
Female	926	842 (90.93)	84 (9.07)	
Education of father, *n* (%)				
High school or below	1451	1244 (85.73)	207 (14.27)	0.531
Junior college or above	413	349 (84.50)	64 (15.50)	
Education of mother, *n* (%)				
High school or below	1444	1234 (85.46)	210 (14.54)	**<0.001**
Junior college or above	420	359 (85.48)	61 (14.52)	
Boarding, *n* (%)				
Yes	803	710 (88.42)	93 (11.58)	**0.002**
No	1061	883 (83.22)	178 (16.78)	
Moderate-to-high-intensity exercise, *n* (%)				
<3 times/week	765	659 (86.14)	106 (13.86)	0.486
≥3 times/week	1099	934 (84.99)	165 (15.01)	
Screen time, *n* (%)				
≤2 h/day	1211	1028 (84.89)	183 (15.11)	0.339
>2 h/day	653	565 (86.52)	88 (13.48)	
Dietary preferences, *n* (%)				
Health group	1550	1324 (85.42)	226 (14.58)	0.909
Unhealthy group	314	269 (85.67)	45 (14.33)	
Nutrition knowledge level, *n* (%)				
Unqualified	770	634 (82.34)	136 (17.66)	**<0.001**
Qualified	1094	959 (87.66)	135 (12.34)	

Note: Categorical variables are presented as the number of cases (%). The analysis involved the use of the chi-square test and chi-square trend test. The bold *p*-value represents “<0.05”.

**Table 2:** Factor loadings and patterns of SSBs for nine types.

SSBs Type	Plant Protein Pattern	Dairy-Containing Pattern	Coffee Pattern
plant protein beverages	0.739		
fruit and vegetable juices and their beverages	0.657		
milk tea beverages	0.580		
tea (and its types) beverages			
dairy-containing beverages		0.757	
plant-based beverages		0.707	
sports beverages			
coffee (and its types) beverages			0.704
carbonated beverages			0.505

Note: The analysis utilized PCA (principal component analysis). Factor loadings below an absolute value of 0.5 were excluded for simplification purposes.

**Table 3:** Model fitting indexes of different classes.

Classes	AIC	BIC	ABIC	LMR	BLRT	Entropy
1	12,974.372	13,018.616	12,993.200			
2	12,579.228	12,673.246	12,619.237	0.0000	0.0000	0.675
3	12,407.414	12,551.206	12,468.605	0.0000	0.0000	0.545
4	12,380.110	12,573.677	12,462.483	0.0594	0.0000	0.668
5	12,369.820	12,613.162	12,473.374	0.1139	0.0000	0.700

Note: The analysis utilized LCA (latent class analysis). AlC is the Aikake information criterion; BIC is the Bayesian information criterion; aBIC is adjusted BIC; LMR is lo-Mendel-Rubin adjusted likelihood; BLRT is the likelihood ratio test based on Bootstrap; entropy is the average information content.

**Table 4:** Characteristics of SSBs pattern score quartiles (Q) in the study participants.

Variable	Plant Protein Pattern		*p*	Dairy-Containing Pattern		*p*	Coffee Pattern		*p*
	Q1	Q4		Q1	Q4		Q1	Q4	
Age, *n* (%)									
9–10	116 (64.09)	65 (35.91)	**<0.001**	75 (45.73)	89 (54.27)	0.232	106 (68.83)	48 (31.17)	**<0.001**
11–13	219 (48.13)	236 (51.87)		228 (49.14)	236 (50.86)		225 (49.45)	230 (50.55)	
14–17	131 (44.26)	165 (55.74)		163 (53.62)	141 (46.38)		135 (41.80)	188 (58.20)	
Sex, *n* (%)									
Male	229 (47.31)	255 (52.69)	0.088	184 (38.10)	299 (61.90)	**<0.001**	209 (41.80)	291 (58.20)	**<0.001**
Female	237 (52.90)	211 (47.10)		282 (62.81)	167 (37.19)		257 (59.49)	175 (40.51)	
Education of father, *n* (%)									
High school or below	360 (49.32)	370 (50.68)	0.427	363 (49.05)	377 (50.95)	0.257	367 (50.41)	361 (49.59)	0.635
Junior college or above	106 (52.48)	96 (47.52)		103 (53.65)	89 (46.35)		99 (48.53)	105 (51.47)	
Education of mother, *n* (%)									
High school or below	366 (49.93)	367 (50.07)	0.936	366 (49.66)	371 (50.34)	0.687	374 (51.52)	352 (48.48)	0.082
Junior college or above	100 (50.25)	99 (49.75)		100 (51.28)	95 (48.72)		92 (44.66)	114 (55.34)	
Boarding, *n* (%)									
Yes	158 (40.93)	228 (59.07)	**<0.001**	200 (50.13)	199 (49.87)	0.947	207 (47.59)	228 (52.41)	0.168
No	308 (56.41)	238 (43.59)		266 (49.91)	267 (50.09)		259 (52.11)	238 (47.89)	
Moderate-to-high-intensity exercise, *n* (%)									
<3 times/week	205 (52.84)	183 (47.16)	0.144	211 (55.97)	166 (44.03)	**0.003**	197 (54.87)	162 (45.13)	**0.018**
≥3 times/week	261 (47.98)	283 (52.02)		255 (45.95)	300 (54.05)		269 (46.95)	304 (53.05)	
Screen time, *n* (%)									
≤2 h/day	313 (52.17)	287 (47.83)	0.075	298 (51.83)	277 (48.17)	0.157	343 (57.07)	258 (42.93)	**<0.001**
>2 h/day	153 (46.08)	179 (53.92)		168 (47.06)	189 (52.94)		123 (37.16)	208 (62.84)	
Dietary preferences, *n* (%)									
Healthy group	411 (53.80)	353 (46.20)	**<0.001**	398 (53.00)	353 (47.00)	**<0.001**	400 (52.63)	360 (47.37)	**0.001**
Unhealthy group	55 (32.74)	113 (67.26)		68 (37.57)	113 (62.43)		66 (38.37)	106 (61.63)	
Nutrition knowledge level, *n* (%)									
Unqualified	190 (50.40)	187 (49.60)	0.841	193 (48.61)	204 (51.39)	0.466	199 (53.07)	176 (46.93)	0.124
Qualified	276 (49.73)	279 (50.27)		273 (51.03)	262 (48.97)		267 (47.94)	290 (52.06)	

Note: The categorical variables are presented as counts (%) in the data. The chi-square trend test and chi-square test were utilized. The bold *p*-value (<0.05) indicates significance.

**Table 5:** Association analysis between SSBs consumption patterns and undernutrition.

SSBs Pattern	Normal	Undernutrition	*p*	Model 1	*p*	Model 2	*p*
				PR (95% CI)		PR (95% CI)	
Plant Protein Pattern, *n* (%)							
Q1	404 (86.70)	62 (13.30)	0.304	1		1	
Q2	395 (84.76)	71 (15.24)		1.145 (0.835, 1.570)	0.400	1.197 (0.878, 1.633)	0.256
Q3	405 (86.91)	61 (13.09)		0.984 (0.708, 1.368)	0.923	1.053 (0.760, 1.459)	0.756
Q4	389 (83.48)	77 (16.52)		1.145 (0.835, 1.570)	0.169	1.302 (0.958, 1.771)	0.092
Dairy-Containing Pattern, *n* (%)							
Q1	417 (89.48)	49 (10.52)	**<0.001 ^a^**	1		1	
Q2	405 (86.91)	61 (13.09)		1.245 (0.874, 1.773)	0.224	1.160 (0.818, 1.644)	0.406
Q3	392 (84.12)	74 (15.88)		1.510 (1.078, 2.116)	**0.017**	1.410 (1.010, 1.968)	**0.043**
Q4	379 (81.33)	87 (18.67)		1.776 (1.282, 2.459)	**0.001**	1.506 (1.088, 2.083)	**0.014**
Coffee Pattern, *n* (%)							
Q1	400 (85.84)	66 (14.16)	0.252	1		1	
Q2	403 (86.48)	63 (13.52)		0.955 (0.693, 1.315)	0.776	0.942 (0.688, 1.290)	0.711
Q3	403 (86.48)	63 (13.52)		0.955 (0.693, 1.315)	0.776	0.951 (0.693, 1.303)	0.753
Q4	387 (83.05)	79 (16.95)		1.197 (0.886, 1.617)	0.241	1.148 (0.849, 1.553)	0.369

Note: Analysis involved the utilization of the chi-square trend test and log-binomial regression. Q1 was designated as the reference group. Model 1 remained unadjusted, while Model 2 was adjusted for gender, age, mother’s education level, boarding status, dietary preferences, and nutritional knowledge level. ^a^ Pearson’s R = 0.086, *p* < 0.001 (a detailed table is in the Supplementary Materials). The bold *p*-value means “<0.05”.

## Data Availability

The data are not publicly available due to privacy. The data presented in the analyses for this study are available on request from the corresponding author.

## Supplementary Materials

The following supporting information can be downloaded at: https://www.mdpi.com/article/10.3390/nu16050650/s1, Table S1: Description of log-binomial regression assignment; Table S2: Symmetric Measures; Table S3: Association analysis for boys between SSBs consumption patterns and undernutrition; Table S4: Association analysis for girls between SSBs consumption patterns and undernutrition; Table S5: Analysis of children aged 9–10 years between SSBs consumption patterns and undernutrition; Table S6: Analysis of children aged 11–13 years between SSBs consumption patterns and undernutrition; Table S7: Analysis of children aged 14–17 years between SSBs consumption patterns and undernutrition.
