# Supplementary material for: Consumption Patterns of Sugar-Sweetened Beverages and Association with Undernutrition among Children Aged 9–17 Years in Guangzhou, China: A Cross-Sectional Study
Source: Nutrients. 2024 Feb 26;16(5):650. doi: 10.3390/nu16050650 (PMC10935377; doi:10.3390/nu16050650)
Supplement: Supplementary file 1 [file nutrients-16-00650-s001.zip › nutrients-2853016-supplementary.pdf]

Supplementary Materials:

Table S1: Description of Log-binomial regression assignment

Factors	Variable	Explain
Undernutrition	Y	Normal=0; undernutrition=1;
SSBs pattern quartiles group	X1	Q2=1；Q3=2；Q4=3；Q1=4；
Age	X2	9-10 years=1; 11-13 years=2; 14-17 years=3;
Gender	X3	Male=1; Female=2;
Boarding status	X4	Yes=1; No=2；
Mother education level	X5	High school or below=1; Junior college or above=2;
Dietary preferences	X7	Healthy group=1；Unhealthy group=2；
Nutrition knowledge level	X8	Unqualified=1;Qualified=2

Table S2:Symmetric Measures

		Value	Asymptotic Standard Error[a]	Approximate T[b]	Approximate Significance
Interval by Interval	Pearson's R	.086	.023	3.774	0.000[c]
Ordinal by Ordinal	Spearman Correlation	.086	.023	3.774	0.000[c]
N of Valid Cases		1864			

Note:a. Not assuming the null hypothesis.b. Using the asymptotic standard error assuming the null hypothesis.c. Based on normal approximation.

Table S3:Association analysis for boys between SSBs consumption patterns and undernutrition

SSBs Pattern	Model (PR 95% CI)	P
Plant Protein Pattern		
Q1	1	
Q2	1.312（0.904，1.904）	0.152
Q3	1.121（0.759，1.656）	0.566
Q4	1.297（0.895，1.878）	0.170
Dairy-containing Pattern		
Q1	1	
Q2	1.117（0.705，1.770）	0.636
Q3	1.509（0.991，2.297）	0.055
Q4	1.654（1.108，2.469）	0.014
Coffee Pattern		
Q1	1	

Q2	0.768（0.510，1.159）	0.209
Q3	0.986（0.684，1.422）	0.940
Q4	1.137（0.806，1.606）	0.464

Note: Log-binomial regression was used to analysis. Q1 was designated as the reference group. Model was adjusted for age, mother's education level, boarding status, dietary preferences, and nutritional knowledge level.The bold *p*-value means "< 0.05".

Table S4: Association analysis for girls between SSBs consumption patterns and undernutrition.

SSBs Pattern	Model (PR 95% CI)	*P*
Plant Protein Pattern		
Q1	1	
Q2	1.022（0.578，1.806）	0.940
Q3	0.900（0.495，1.634）	0.729
Q4	1.377（0.785，2.414）	0.264
Dairy-containing Pattern		
Q1	1	
Q2	1.274（0.743，2.185）	0.379
Q3	1.293（0.731，2.287）	0.378
Q4	1.090（0.579，2.051）	0.789
Coffee Pattern		
Q1	1	
Q2	1.245（0.741，2.092）	0.408
Q3	0.790（0.423，1.473）	0.458
Q4	1.086（0.574，2.056）	0.799

Note: Log-binomial regression was used to analysis. Q1 was designated as the reference group. Model was adjusted for age, mother's education level, boarding status, dietary preferences, and nutritional knowledge level.

Table S5: Analysis of children aged 9-10 years between SSBs consumption patterns and undernutrition.

SSBs Pattern	Model (PR 95% CI)	*P*
Plant Protein Pattern		
Q1	1	
Q2	1.194（0.652，2.184）	0.566
Q3	1.025（0.517，2.033）	0.944
Q4	1.901（1.091，3.313）	**0.023**
Dairy-containing Pattern		
Q1	1	
Q2	1.008（0.510，1.993）	0.981
Q3	1.282（0.655，2.507）	0.468
Q4	1.441（0.770，2.700）	0.253
Coffee Pattern		
Q1	1	

Q2	1.372（0.785，2.399）	0.267
Q3	0.893（0.432，1.845）	0.759
Q4	1.873（1.018，3.446）	**0.044**

Note: Log-binomial regression was used to analysis. Q1 was designated as the reference group. Model was adjusted for gender, mother's education level, boarding status, dietary preferences, and nutritional knowledge level. The bold *p*-value means "< 0.05".

Table S6:Analysis of children aged 11-13 years between SSBs consumption patterns and undernutrition.

SSBs Pattern	Model (PR 95% CI)	*P*
Plant Protein Pattern		
Q1	1	
Q2	1.158（0.752，1.783）	0.506
Q3	0.847（0.524，1.370）	0.499
Q4	0.936（0.588，1.488）	0.799
Dairy-containing Pattern		
Q1	1	
Q2	1.175（0.709，1.946）	0.532
Q3	1.292（0.780，2.139）	0.319
Q4	1.493（0.924，2.412）	0.102
Coffee Pattern		
Q1	1	
Q2	0.718（0.447，1.154）	0.171
Q3	0.776（0.495，1.219）	0.271
Q4	0.933（0.611，1.425）	0.749

Note: Log-binomial regression was used to analysis. Q1 was designated as the reference group. Model was adjusted for gender, mother's education level, boarding status, dietary preferences, and nutritional knowledge level.

Table S7: Analysis of children aged 14-17 years between SSBs consumption patterns and undernutrition.

SSBs Pattern	Model (PR 95% CI)	*P*
Plant Protein Pattern		
Q1	1	
Q2	1.191（0.607，2.336）	0.611
Q3	1.338（0.713，2.510）	0.365
Q4	1.483（0.794，2.769）	0.217
Dairy-containing Pattern		
Q1	1	
Q2	1.218（0.616，2.409）	0.571
Q3	1.638（0.898，2.987）	0.108
Q4	1.577（0.848，2.932）	0.150
Coffee Pattern		
Q1	1	

Q2	0.935（0.469，1.863）	0.848
Q3	1.353（0.736，2.489）	0.331
Q4	1.138（0.622，2.082）	0.675

Note: Log-binomial regression was used to analysis. Q1 was designated as the reference group. Model was adjusted for gender, mother's education level, boarding status, dietary preferences, and nutritional knowledge level.
